# An anti-inflammatory neuroenhancer mitigates amyloid-β pathology to improve Alzheimer's disease therapy

**DOI:** 10.1016/j.mtbio.2026.102874

**Published:** 2026-01-31

**Authors:** Weiqing Fang, Jing Zhao, Li Li, Yu Wang, Zhi Ping Xu, Lingxiao Zhang

**Affiliations:** a Department of Pharmacy, School of Medicine, Women's Hospital, Zhejiang University, Hangzhou, 310023, China; b School of Medicine, Hangzhou City University, Hangzhou, 310015, China; c Institute of Chemical Biology, Shenzhen Bay Laboratory, Shenzhen, 518107, China; d Gulbali Research Institute, Charles Sturt University, Wagga, NSW, 2678, Australia; e Interdisciplinary Nanoscience Center, Aarhus University, Aarhus C, DK-8000, Denmark

**Keywords:** Lipid-coated calcium phosphate nanoparticles, Alzheimer's disease, Natural compounds, siRNA, β-amyloid, Neuroinflammation

## Abstract

β-amyloid (Aβ) inhibition significantly attenuates the early-stage Alzheimer's disease (AD) progression, but the improvement in cognitive function remains limited by neuroinflammation. Here, we developed a bioinspired neuroenhancer that concurrently targets both Aβ aggregation and neuroinflammation. Rutin and small interfering RNA targeting beta-site amyloid precursor protein cleaving enzyme 1 (siBACE1) were co-loaded into the calcium phosphate core, which was further coated with lipid bilayers and Angiopep-2/rabies virus glycoprotein 29 peptides to form the multifunctional neuroenhancer (RB@LCP-AR). RB@LCP-AR not only releases siBACE1 to silence BACE1 expression and block Aβ production from the cleavage of amyloid precursor protein, but also releases Rutin to suppress the Aβ aggregation. Moreover, the released Rutin of RB@LCP-AR directly alleviates Aβ-induced mitochondria dysfunction and intracellular ROS production in neuronal cells. Notably, the targeting of RB@LCP-AR to neurons and the inhibition of Aβ reduce the microgliosis and astrogliosis, further alleviating neuroinflammation and synapse loss. Consequently, AD mice receiving RB@LCP-AR treatment efficiently recovered their memory and cognition. Our study thus provides a coordinated targeting of Aβ and neuroinflammation inhibition, holding considerable potential to promote the recovery of memory and cognition in AD.

## Graphical abstract

A dual-functional bioinspired neuroenhancer RB@LCP-AR reduces amyloid-β burden and while eliminating neuroinflammation to rescue memory and cognition loss in Alzheimer's disease mice.

**Figure:**
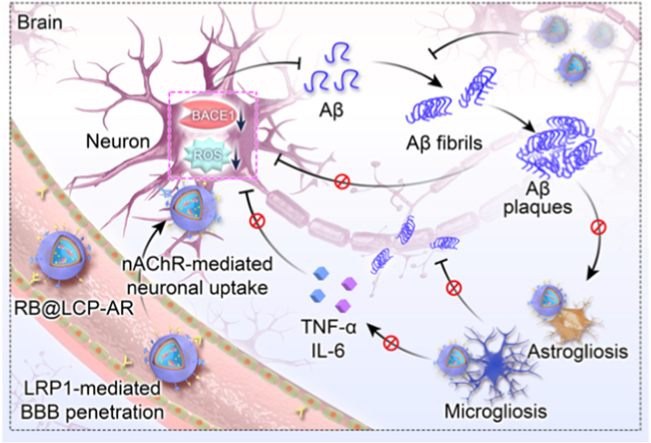

## 1 Introduction

Alzheimer's disease (AD) is a progressive neurodegenerative disorder that affects more than 33 million individuals worldwide [1]. β-amyloid (Aβ), generated by β-site amyloid precursor protein cleaving enzyme 1 (BACE1)-mediated cleavage of amyloid precursor protein (APP), is considered an early pathogenic factor, with abnormal accumulation beginning 15–20 years before clinical diagnosis [[2], [3], [4]]. Aβ aggregation produces toxic oligomers (Aβo) that impair mitochondrial function and cause neuronal cytotoxicity, eventually assembling into fibrils and plaques deposited in the brain [5]. Although Aβ clearance has long been a therapeutic focus, most Aβ-targeted approaches have failed or been discontinued due to adverse effects [6]. Recently, the Food and Drug Administration (FDA) approved the monoclonal antibodies (aducanumab, donanemab, and lecanemab), which have shown efficacy in reducing Aβ burden and slowing early-stage disease progression [7,8]. However, significant safety concerns remain with the single-target therapeutic strategies, as evidenced by the donanemab Phase III trial, where nearly 25% of participants developed amyloid-related imaging abnormalities and three deaths occurred [9]. These adverse effects are largely attributed to antibody-induced neuroinflammation and excessive synaptic pruning [10,11]. In addition, chronic exposure to Aβ aggregates drives microglial overactivation, further amplifying neuroinflammation and excessive synaptic pruning [12]. Therefore, multiple-target therapeutic strategies that simultaneously reduce Aβ burden and alleviate neuroinflammation are critical for the development of next-generation AD treatments. Numerous studies have demonstrated that co-assembling Aβ-inhibiting motifs with anti-inflammatory components into bifunctional therapeutic agents can significantly improve behavioral and memory performance in AD mice [13,14]. However, given the complex pathological mechanisms and significant patient heterogeneity in AD [15], the targeting of Aβ-reducing and anti-inflammatory agents may lack the adaptability required for broader clinical application [16]. Consequently, a modular delivery platform capable of flexibly adjusting therapeutic components represents a promising strategy to achieve more precise and individualized intervention.

Various modular delivery platform, including supramolecular [16], albumin [17], albumin/nano-aluminum adjuvant [18], membrane-coated hollow mesoporous Prussian blue nanoparticles [19], and poly(lactic-co-glycolic acid) [20], have been explored for delivering functional agents in AD mice. Notably, calcium phosphate (CaP) emerges as a unique and promising candidate due to its inherent biosafety and biomimetic properties. Already approved by the FDA for bone regeneration and repair, CaP represents an ideal platform for delivering therapeutic components. This efficacy stems from its nature as a bioinspired material, whose composition and structure directly mimic those of natural bone and teeth mineral in mammalian [[21], [22], [23]]. Lipid-coated calcium phosphate (LCP), an advanced formulation, offers improved colloidal stability and biocompatibility [24]. Once internalized, LCP rapidly dissociates within acidic organelles, enabling efficient cytosolic release of loaded cargoes [25]. To validate this platform, we selected Aβ production and oxidative stress as two representative targets. Different from antibody-mediated Aβ clearance, BACE1 siRNAs have effectively ameliorated AD neuropathology by reducing Aβ production in transgenic models [26]. Rutin, a natural compound derived from edible plants, also exerts neuroprotective effects in AD models by inhibiting Aβ aggregation, alleviating neuroinflammation, and enhancing neuronal survival without apparent side effects [27,28]. However, limited bioavailability remains a major obstacle for both BACE inhibitors and natural compounds [29,30]. Fortunately, blood-brain barrier (BBB) breakdown often precedes the onset of AD symptoms and continues throughout disease progression [31], creating opportunities for nanoplatforms to enhance brain delivery of such therapeutics [32]. LCP may significantly enhance the therapeutic utilization of Rutin. Likewise, siRNAs can be readily encapsulated into CaP *via* electrostatic interactions and subsequently released into the cytosol [33].

**Fig. 1:**
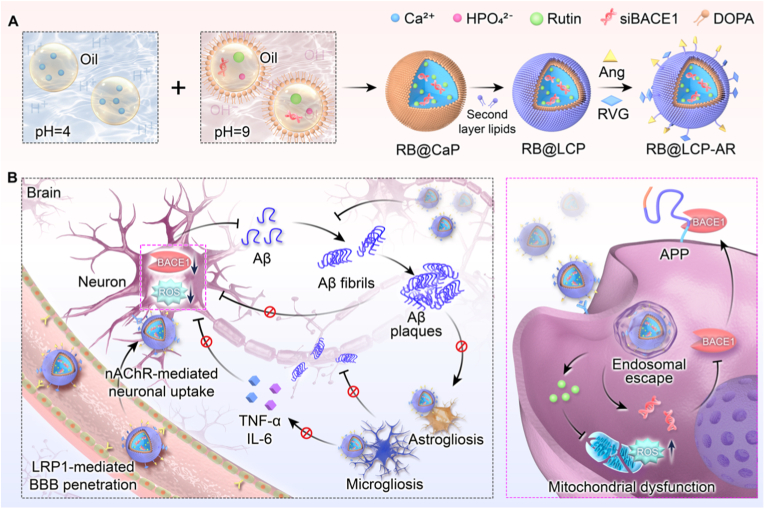
Schematic illustration of the design and function of bioinspired neuroenhancer (RB@LCP-AR). (A) RB@LCP-AR was synthesized by a water-in-oil micro-emulsion method, which loaded with Rutin and siBACE1, and coated with lipid layers and Ang/RVG peptides. (B) The neuroenhancer effectively crosses the BBB and specifically targets neurons. Upon endocytosis endosomal escape and disassembly, Rutin and siBACE1 were released to inhibit Aβ and reduce intracellular ROS production. In brain tissues, the targeting of RB@LCP-AR to neurons and the inhibition of Aβ reduce the microgliosis and astrogliosis, further alleviating neuroinflammation and synapse loss.

In this study, we developed a bioinspired neuroenhancer based on a CaP modular delivery platform to simultaneously reduce Aβ burden and neuroinflammation for enhanced AD therapy. Specifically, BACE1 siRNA (siBACE1), designed to suppress Aβ production, and the anti-inflammatory compound Rutin were co-loaded into CaP core (RB@CaP) *via* a co-precipitation process. Then the core was coated with layer lipids to obtain LCP nanoparticles (RB@LCP). The nanoparticles were further surface-modified with the endothelial low-density lipoprotein receptor 1 (LRP1) ligand Angiopep-2 (Ang, for BBB penetration [34]) and nicotinic acetylcholine receptor (nAChR) ligand rabies virus glycoprotein 29 (RVG, for neuron targeting [35]) to generate dual-targeting nanoparticle RB@LCP-AR (Fig. 1A). After crossing the BBB, RB@LCP-AR was primarily internalized by neurons, where it rapidly escaped from endosomes and released siBACE1 to inhibit BACE1-mediated Aβ production, along with Rutin to alleviate mitochondrial dysfunction and intracellular reactive oxygen species (ROS) accumulation (Fig. 1B). The fraction of RB@LCP-AR internalized by microglia or astrocytes further attenuated their inflammatory state. Moreover, the inhibition of Aβ reduced the microgliosis and astrogliosis, further alleviating synapse loss. As a result, RB@LCP-AR effectively reduced both Aβ burden and neuroinflammation in the AD brain, ultimately contributing to the recovery of memory and cognition. Overall, this study introduces a new paradigm for AD nanotherapeutics based on FDA-approved materials and agents, offering a promising candidate for effective AD treatment.

## 2 Materials and methods

### 2.1 Materials

The phospholipids including 1,2-dioleoyl-sn-glycero-3-phosphate (DOPA), 1,2-dioleoyl-sn-glycero-3-phosphocholine (DOPC), 1,2-dioleoyl-3-trimethylammonium- propane (DOTAP), 1,2-distearoyl-sn-glycero-3-phosphoethanolamine-N- [methoxy(polyethylene glycol)-2000] (DSPE-PEG2000-OCH3), 1,2-distearoyl-sn-glycero- 3-phosphoethanolamine-N-[maleimide(polyethylene glycol)-2000] (DSPE-PEG2000- Melaimid) were purchased from Avanti Polar Lipid. Ang (TFFYGGSRGKRNNFKTEEYC) and RVG (TWMPENPRPGTPCDIFTNSRGKRASNGGGGGGC) were provided by GL Biochem (Shanghai, China). Sulfosuccinimidyl 4-(N-maleimidomethyl) cyclohexane-1- carboxylate (Sulfo-SMCC) and 1,1,1,3,3,3-Hexafluoro-2-propanol (HFIP) were obtained from Thermo Fisher. MTT (3-(4,5-dimethylthiazol-2-yl)-2,5-diphenyltetrazolium bromide) was purchased from Beyotime. Cyanine7 (Cy5) was purchased from Med Chem Express (New Jersey, USA). Mitochondrial Membrane Potential Assay Kit (M8650) with JC-1 and 2′,7′-dichlorofluorescein diacetate (DCFH-DA, ID3130) were purchased from Solarbio (China). All other chemicals and reagents were obtained from Sigma-Aldrich. All materials were used as received with no further purification unless specifically mentioned.

### 2.2 Cell lines and animals

Neuro-2a (N2a) cells and BV2 cells were routinely cultured in DMEM supplemented with 10% fetal bovine serum (FBS) and maintained at 37 °C with 5% CO_2_. All the cell lines were obtained from the China Infrastructure of Cell Line Resources (Beijing, China). AD (APP/PS1) mice were obtained from Model Animal Research Center of Nanjing University.

### 2.3 Synthesis and characterization of nanoparticles

#### 2.3.1 Synthesis of LCP

LCP nanoparticles were fabricated by a modified two-steps method as describe previously with a few modifications [33]. In brief, 150 μL of CaCl_2_ (2.5 M, pH 4.0) was dispersed in a mixture of cyclohexane/Igepal CO-520 (5 mL, V/V = 7/3) and stirred for 20 min to form a well-dispersed microemulsion. Another microemulsion solution containing 150 μL of Na_2_HPO_4_ (25 mM, pH 9.0) was prepared in a similar way, followed by replenishing of 1 μmol of DOPA under magnetic stirring. The second microemulsion solution was added dropwise to the first one to make DOPA-coated CaP cores. After 20 min, the cores were centrifuged and washed with absolute ethanol. The core pellets were dispersed in 1 mL chloroform with a total of 1.4 μmol of second layer lipids (1% DOTAP, 10% DSPE-PEG2000-OCH_3_, 10% DSPE-PEG2000-Maleimide, 29% DOPC, and 50% cholesterol) with ultrasonic. The chloroform was then evaporated using a rotovap. The lipid film was then hydrated in PBS buffer (pH = 7.4) to obtain LCP nanoparticles.

#### 2.3.2 Synthesis of Rutin-loaded LCP (R@LCP)

Similarly, CaCl_2_ (2.5 M, 150 μL) with pH adjusted to 4.0–6.0 was dispersed in a cyclohexane/Igepal CO-520 mixture (5 mL) to form a well-dispersed microemulsion. To prepare R@LCP, 0.75 mg of Rutin (dissolved in 0.03 M NaOH) was added to the Na_2_HPO_4_ solution. The Na_2_HPO_4_ solution was then used to co-precipitate CaP cores, which were subsequently coated with DOPA. After collection and redispersion in chloroform, DOPA-coated CaP cores were further coated with the second layer lipids (1% DOTAP, 10% DSPE-PEG2000-OCH_3_, 10% DSPE-PEG2000-Maleimide, 29% DOPC, and 50% cholesterol). The corresponding R@LCP products were obtained after chloroform evaporation, using CaCl_2_ solution at varying pH values (4.0–6.0).

#### 2.3.3 Synthesis of LCP containing siBACE1 (B@LCP) and RB@LCP

To synthesize B@LCP, 3 nmol of siBACE1 were added to the Na_2_HPO_4_ solution prior to CaP core formation. For the preparation of dual-loaded RB@LCP nanoparticles, both Rutin and siBACE1 were simultaneously incorporated into the Na_2_HPO_4_ solution.

#### 2.3.4 Maleimide conjugation of Ang/RVG peptides

The Ang/RVG peptide solution (10 mg mL^−1^ in dimethyl sulfoxide, DMSO) was mixed with equal volume of 100 mM tris(2-carboxyethyl) phosphine hydrochloride (TCEP) and stirred for 30 min. The resultant solution was mixed with 10 mg mL^−1^ DSPE-PEG2000-maleimide solution in DMSO and stirred for 2 h with continuous purging of N_2_ followed by dialysis to remove unconjugated complexes. Subsequently, R@LCP-AR, B@LCP-AR, and RB@LCP-AR were obtained by coating the core pellets with a second lipid layer composed of 1% DOTAP, 10% DSPE-PEG2000-OCH_3_, 5% DSPE-PEG_2000_-Maleimide-RVG, 5% DSPE-PEG2000-Maleimide-Ang, 29% DOPC, and 50% cholesterol. The peptide loading efficiency was calculated by measuring the decrease in peptide concentration in suspension using BCA assay.

#### 2.3.5 Characterization of nanoparticles

The resultant nanoparticles were characterized using dynamic light scattering (DLS) to determine their hydrodynamic size and zeta potential (Nanosizer, Malvern, UK). Transmission electron microscopy (TEM) images were obtained using a Zeol TEM (JEM-3010, ZEOL, Tokyo, Japan) and the size distribution of nanoparticles was analyzed using NanoMeasurer. The amount of Rutin loaded onto LCPs was determined by measuring UV–Vis absorbance at 265 nm.

### 2.4 Cellular uptake assay

N2a or BV2 cells were seeded in glass-bottom confocal dishes (1 × 10^5^ cells per well) and incubated overnight. Cells were then treated with Saline, Cy5-siBACE1, Cy5-RB@LCP, or Cy5-RB@LCP-AR (Cy5 equivalent concentration: 40 nM) for 24 h. After incubation, cells were stained with LysoTracker Red (Beyotime, China), fixed with 4% paraformaldehyde, and stained the nuclei with DAPI. Confocal images were obtained using a confocal laser scanning microscope (Zeiss LSM 880). In another assay, N2a or BV2 cells were seeded in a 24-well plate (5 × 10^4^ cells per well). Subsequently, the medium was then replaced with Cy5-siBACE1, Cy5-RB@LCP, or Cy5-RB@LCP-AR-containing medium and incubated for 24 h. The cells were then harvested for flow cytometry analysis using CytoFLEX instrument (Beckman).

To mimic the BBB penetration *in vitro*, b.End3 cells (2 × 10^5^ cells per well) were seeded in the upper chambers (PET membrane, 0.4 μm pore size, Falcon; Fisher Scientific) while N2a cells (recipients) were plated at a density of 5 × 10^5^ cells in a 12-well plate. The particles were added to the above chamber and incubated for 4 or 24 h. Then the N2a cells were harvested and the proportion of positive cells were analyzed by flow cytometry.

### 2.5 Analysis of gene expression

N2a or BV2 cells were seeded in a 24-well plate (5 × 10^4^ cells per well). After 8 h, the medium was replaced with siBACE1, RB@LCP, or RB@LCP-AR-containing medium and incubated for 24 h. Total RNA was extracted from cells using commercial RNA extraction kit (Vazyme, China). Then the expression level of BACE1 was quantified by quantitative real-time PCR (qPCR). BACE1 primer sequences were used as previously described [18]. In animal experiments, the brain was harvested after euthanasia and the cortex and hippocampus were separated for BACE1 expression analysis by qPCR. The expression of N2a and b.End3 cells were detected by qPCR as well. To assess nAChR expression in N2a, b.End3, and BV2 cells, total RNA was extracted and quantified by qPCR.

### 2.6 Preparation of Aβ aggregates

One mg of Aβ 1–40/1-42 was dissolved in 1 mL of HFIP and ultrasonicated at room temperature for 10 min to achieve a clear solution. The resultant solution was aliquoted in Eppendorf tubes, dried under vacuum at room temperature and stored at −20 °C. To prepare Aβ oligomer and fiber, 50 μg of HFIP-treated Aβ was completely dissolved in NaOH (50 μL, 50 mM), followed by the addition of chilled PBS. Then the Aβ 42 solution was incubated at room temperature for 2 days to get Aβ oligomer and fiber.

### 2.7 MTT assay

N2a cells were seeded in a 96-well plate at a density of 1 × 10^4^ cells per well and incubated overnight. Then, replace the media with fresh media containing RB@LCP-AR (12.5–400 μg mL^−1^) or Aβ (0.0001–10 μM) and incubate for 24 h. Cell viability was detected by adding 100 μL of 5 mg mL^−1^ MTT to each well and incubation for 4 h at 37 °C. Then, the media was removed and 50 μL DMSO was added to each well and shaken for 10 min to solubilize the formazan crystals. Finally, the absorbance was measured by a Tecan microplate reader and cell viability was determined by calculating the relative absorbance of the samples compared to the absorbance of the media.

To evaluate the mitigative effect, N2a cells were treated with Aβ oligomer for 24 h. Then, the B@LCP-AR, Rutin, or RB@LCP-AR was added to media and incubated for another 24 h to relieve the burden of Aβ oligomer. The mitigative effect was then detected by MTT assay.

### 2.8 ROS assay

N2a cells were incubated with Aβ for 24 h, and then incubated with RB@LCP-AR, B@LCP-AR, or Rutin for 6 h. After treatment, the cells were stained with ROS probe, 20 μM DCFH-DA for 30 min at 37 °C in dark. Fluorescence intensity was then measured using a microplate reader. The relative ROS level was calculated as the fluorescence intensity (λex 485 nm, λem 530 nm) normalized to the untreated group.

### 2.9 Mitochondrial membrane potential assay

Aβ (1 μM)-treated N2a cells were incubated with B@LCP-AR, Rutin, or RB@LCP-AR for 24 h. Then, JC-1 solution was added to cells and stained for 0.5 h, the mitochondrial JC-1 fluorescence intensity of the monomeric (green) and aggregated (red) forms was detected by microplate reader. To obtain the mitochondrial membrane potential, the ratios of red/green were calculated and normalized to Saline-treated group. In a parallel assay, the mitochondrial JC-1 fluorescence intensity in monomer or aggregate form was detected using a confocal laser scanning microscope (Zeiss LSM 880).

### 2.10 Mouse behavioral tests

AD mice (APP/PS1) aged 9 months were injected with saline, R@LCP-AR, B@LCP-AR or RB@LCP-AR (50 mg kg^−1^ per dose) intravenously 5 times at an interval of 4 days (day 0–16), WT mice were served as the negative control. Then the memory and cognition of mice were tested by Morris water maze (MWM), Y-maze and novel object recognition test.

The MWM test was performed in a circular pool with a diameter of 1.2 m filled with opaque water. Mice were trained twice daily, and each trial ended with a 10s stay on the hidden platform (day 20–26). Mouse swimming activity was automatically recorded. On the day following the final training session (day 27), the platform was removed to detect the memory retention on all mice.

Then the Y-maze test was performed to test short-term spatial recognition memory of the mice (day 27–28). In a single 5-min trial, each mouse was allowed to freely explore all 3 arms of the maze. Arm entries were recorded using a CCD camera connected to a computer. An alternation was defined as successive entries into all three arms without repetition. The maximum number of possible alternations was calculated as the total number of arm entries minus 2. The percentage of alternations was calculated as: (actual alternations/maximum alternations) × 100%.

The novel object recognition was performed to detect cognitive function of the mice (day 29). Mice naturally tend to spend more time exploring a novel object than a familiar one. In the habituation phase, each mouse was allowed to explore the open-field area freely (a white box, 40 cm × 40 cm × 40 cm) without any objects. During the familiarization period, each mouse was placed in the same box containing two identical objects for 5 min. After 24 h, recognition memory was tested by replacing one of the familiar objects with a novel one. The time of mice spent to explore and sniff each object was recorded.

### 2.11 Aβ40/42, cytokine, oxidative damage marker, and superoxide dismutase (SOD) detection

All mice were euthanized after completion of behavioral tests for brain tissue collection. Homogenization of the right hemisphere was performed in RIPA buffer to obtain the protein extract, followed by centrifugation at 4 °C (14,000 g, 30 min). The levels of Aβ42 and Aβ40 in RIPA-soluble and -insoluble fractions were quantified by ELISA kits and normalized to brain tissue weight. Then the levels of tumor necrosis factor-α (TNF-α) and interleukin-6 (IL-6) in the supernatant of brain lysates were also detected using ELISA kits (Neobioscience technology, China). The supernatant absorbance was measured using multi-detection microplate reader (Aglient, USA).

The concentration of oxidative damage marker malondialdehyde (MDA) in brain tissues was detected using thiobarbituric acid (TBA) test [36]. The characteristic absorbance at 532 nm of the MDA-TBA2 adduct, formed by the reaction of MDA with TBA, was measured using a microplate reader (Agilent, USA).

The SOD activity was detected by SOD assay kit (Beyotime, China). In brief, the enzyme working solution, and nitroblue tetrazolium were added to the prepared brain lysate, and then incubated for 20 min at 37 °C. The absorbance was measured at 560 nm using a microplate reader (Aglient, USA).

### 2.12 Immunohistochemistry

The left hemisphere was processed into paraffin sections for immunohistochemistry. After deparaffinization and rehydration, antigen retrieval was performed, followed by incubation with endogenous peroxidase blocking reagent (ZSGB-BIO, China) for 10 min. Sections were then incubated with the primary antibody against ionized calcium-binding adaptor molecule-1 (Iba-1) and glial fibrillary acidic protein (GFAP) for 60 min, respectively. Next, the sections were incubated with HRP-conjugated goat anti-mouse/rabbit IgG polymer for 20 min. To detect the synaptic protein, the sections were incubated with the primary antibody against synaptophysin and PSD95, and then incubated with corresponding secondary antibodies conjugated to Alexa Fluor 488 (Santa Cruz Biotechnology, USA) or Alexa Fluor 594 (Santa Cruz Biotechnology, USA), respectively.

### 2.13 Statistical analysis

Data are presented as mean ± SEM from at least three independent experiments. Statistical analysis was conducted by Graphpad Prism 9 software *via* a *t*-test. A p value < 0.05 was considered statistically significant. ∗*p* < 0.05, ∗∗*p* < 0.01, ∗∗∗*p* < 0.001, ∗∗∗∗*p* < 0.0001.

## 3 Results and discussion

### 3.1 Characteristics of RB@LCP-AR

**Fig. 2:**
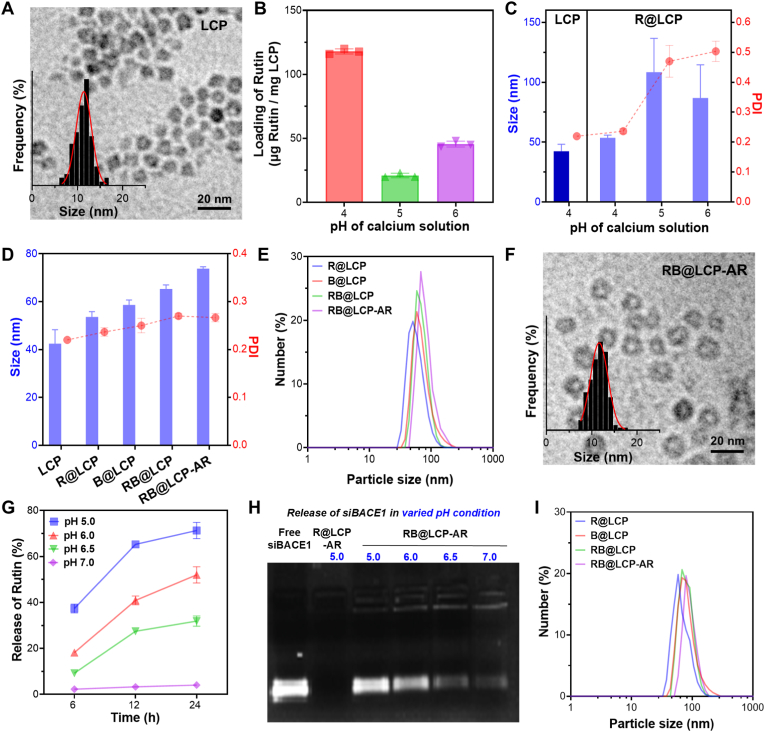
Characterization of RB@LCP-AR. (A) TEM image of LCP. Core size distribution (inset) was analyzed using NanoMeasurer (count = 150). (B) The loading efficiency of Rutin by LCP. (C) The hydrodynamic particle sizes of LCP and R@LCP were prepared with varying pH values of calcium solutions. (D) The hydrodynamic particle size and polydispersity (PDI) of the indicated formulations. (E) The hydrodynamic particle size distribution of the indicated formulations dispersed in FBS after 24 h. (F) TEM image of RB@LCP-AR. Core size distribution (inset) was analyzed using NanoMeasurer (count = 150). (G–H) The release of Rutin (G) and siBACE1 (H) from RB@LCP-AR in pH varied buffers. (I) The hydrodynamic particle size distribution of the indicated formulations dispersed in saline for one week. Data are presented as mean ± SEM.

LCP nanoparticles, consisting of Ca^2+^ and HPO_4_^2−^ core precipitates coated with lipid bilayers, were successfully prepared, as described in our previous studies [33,37]. TEM images confirmed that the LCP cores exhibited a uniform size of 11.4 ± 1.7 nm (Fig. 2A). Rutin and siBACE1 were encapsulated into the cores by pre-dispersing them in Na_2_HPO_4_ solution, followed by emulsification in a cyclohexane/Igepal CO-520 mixture to form a water-in-oil microemulsion, and then co-precipitation with CaCl_2_-containing microemulsion. The pH of the CaCl_2_ solution was found to be a critical factor influencing both Rutin loading efficiency and particle size of the resulting R@LCP [38]. At pH 4.0 of the CaCl_2_ solution, the lowest core pH condition tested, co-precipitation of calcium-Rutin and CaP was promoted, leading to enhanced Rutin loading. As shown in Fig. 2B and C, Rutin loading efficiency in this condition reached 118 μg per mg LCP, with a hydrodynamic particle size of 53.6 nm, slightly larger than pristine LCP (42.5 nm). In contrast, increasing the CaCl_2_ solution pH markedly reduced Rutin loading and caused unstable particle formation, with hydrodynamic sizes of 108.4 and 86.8 nm at pH 5.0 and 6.0, respectively. Thus, pH 4.0 seemed optimal and was selected for subsequent experiments. Under this similar condition, siBACE1 was successfully loaded into LCP cores, yielding B@LCP and dual-loaded RB@LCP with the particle size of 60.6 and 66.8 nm, respectively.

To achieve dual targeting of the BBB and neurons, Ang and RVG peptides were conjugated onto RB@LCP surfaces *via* DSPE-PEG2000-maleimide. Based on peptide concentration changes before and after conjugation, each mg of RB@LCP-AR was found to contain ∼24 μg of Ang and ∼34 μg of RVG (Table S1), and surface modification increased the hydrodynamic particle size of RB@LCP-AR to 74.4 nm (Fig. 2D). All particles maintained excellent colloidal stability after 24 h in serum (Fig. 2D and E), attributable to lipid surface coatings [39]. TEM further showed that RB@LCP-AR cores remained uniform with the average size of 11.7 ± 1.9 nm (Fig. 2F), smaller than the hydrodynamic diameter due to poor lipid boundary resolution in TEM [37]. Drug release studies confirmed acid-responsive behavior. Minimal Rutin release occurred in pH 7.0 buffer over 24 h, whereas ∼70% release was observed in pH 5.0 buffer mimicking lysosomal conditions (Fig. 2G). Similarly, siBACE1 release was effective in buffers of pH 6.0 or lower after 6 h (Fig. 2H). Storage stability was also confirmed, as nanoparticles dispersed in saline and stored at 4 °C for one week showed only a slight size increase (∼10 nm) due to mild aggregation, while maintaining a narrow particle size distribution, indicative of a largely monodisperse state (Fig. 2I).

### 3.2 RB@LCP-AR reduces neuronal BACE1 expression and mitochondrial dysfunction

**Fig. 3:**
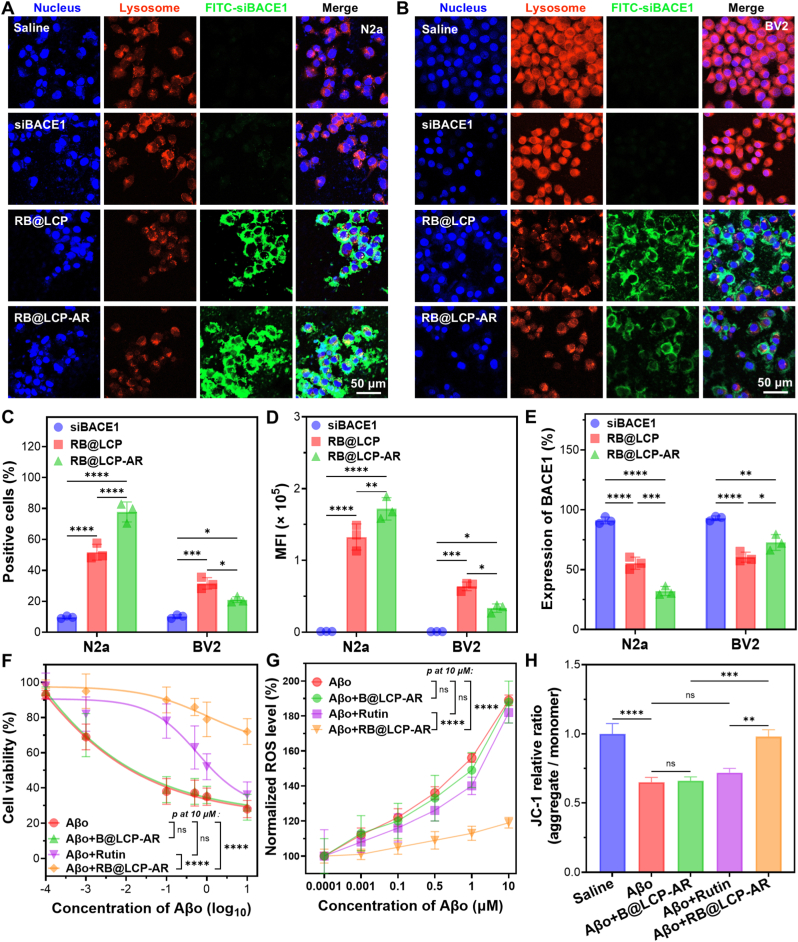
Gene silence and anti-inflammation efficacy of RB@LCP-AR. (A–B) Cellular distribution of RB@LCP and RB@LCP-AR in N2a (A) and BV2 (B) cells after co-incubation for 24 h. (C–D) Cellular uptake of RB@LCP and RB@LCP-AR by N2a and BV2 cells, the proportion of positive cells (C) and MFI (D) of nanoparticles was calculated. (E) BACE1 mRNA expression in N2a and BV2 cells after 24 h treatment with RB@LCP and RB@LCP-AR. (F–G) Cell viability (F) and intracellular ROS level (G) of N2a cells treated with Aβo (0.001–10 μM) and the indicated formulations (Rutin 11.8 μg mL^−1^, B@LCP-AR or RB@LCP-AR: 100 μg mL^−1^). (H) N2a cells treated with Aβo (1 μM) and the indicated formulations (Rutin 11.8 μg mL^−1^, B@LCP-AR or RB@LCP-AR: 100 μg mL^−1^) were stained with JC-1, then the ratio of JC-1 aggregate, and monomer was analyzed by microplate reader. Data are presented as mean ± SEM. ∗*p* < 0.05, ∗∗*p* < 0.01, ∗∗∗*p* < 0.001, ∗∗∗∗*p* < 0.0001.

As shown in Fig. 3A, cellular uptake analysis demonstrated that RB@LCP was readily internalized by N2a neuronal cells after 24 h of incubation. At this time point, most particles escaped into the cytoplasm (non-overlapping green fluorescence), with only a small fraction remaining in lysosomes (yellow fluorescence). Compared with RB@LCP, significantly more RB@LCP-AR was internalized by N2a cells, evidenced by stronger green fluorescence, largely due to RVG surface modification, which specifically recognizes neuronal cells [18,40]. Similarly, both RB@LCP and RB@LCP-AR were internalized by BV2 microglia and subsequently escaped from endosomes/lysosomes. Notably, RB@LCP-AR showed preferential neuronal uptake over microglial uptake, attributed to DSPE-PEG surface coating, which reduces nonspecific serum protein adsorption and macrophage recognition [41] and enhanced targeting by RVG modification (Fig. 3B). This was supported by flow cytometry, which showed much more LCP-positive N2a cells compared with BV2 cells under the same treatment, along with much higher nanoparticle mean fluorescence intensity (MFI), particularly in RB@LCP-AR-treated cells (Fig. 3C and D).

Next, the gene-silencing effect of RB@LCP-AR on BACE1 was examined. Benefiting from neuron-specific delivery, RB@LCP-AR achieved the most efficient BACE1 knockdown in N2a cells, reducing BACE1 mRNA to ∼1/3 and 3/5 of the level observed with free siBACE1 and RB@LCP, respectively (Fig. 3E). In BV2 cells, RB@LCP treatment also reduced BACE1 expression, even more efficient than RB@LCP-AR, probably due to more particle internalization (Fig. 3D and E). These findings indicate that RVG modification enables RB@LCP-AR to be preferentially recognized and internalized by neuronal cells, thereby enhancing precision of siBACE1 delivery, just to neuron cells. Moreover, MTT assays showed that RB@LCP-AR exhibited low cytotoxicity in both N2a and BV2 cells (Fig. S1A).

On this basis, the anti-inflammatory efficacy of RB@LCP-AR was investigated. Aβo showed potent cytotoxicity in N2a cells at concentrations above 0.1 μM (Fig. S1B), consistent with our previous study [18]. As shown in Fig. 3F–B@LCP-AR failed to rescue N2a cell viability under Aβo treatment. Free Rutin displayed some protective effects at concentrations above 0.1 μM, whereas RB@LCP-AR effectively delivered Rutin into N2a cells, restoring cell viability to 70%∼80% even at 10 μM of Aβo. ROS analysis further demonstrated that RB@LCP-AR significantly reduced Aβo-induced ROS production, while free Rutin or B@LCP-AR had no appreciable effect (Fig. 3G). Elevated ROS in neuronal cells is closely linked to Aβo-induced mitochondrial dysfunction [42]. Indeed, Aβo alone markedly reduced the JC-1 aggregate/monomer ratio, indicating severe mitochondrial damage (Fig. 3H). Neither B@LCP-AR nor free Rutin improved mitochondrial function, due to insufficient anti-inflammatory capacity or poor bioavailability of Rutin. In contrast, RB@LCP-AR restored the JC-1 aggregate/monomer ratio to the level comparable with the saline group, demonstrating that intracellular delivery of Rutin *via* LCP nanoparticles effectively alleviated Aβo-induced mitochondrial dysfunction (Fig. 3H, Fig. S2).

Taken together, these results confirm that RVG modification enables RB@LCP-AR to preferentially target neuronal cells, where it silences BACE1 expression and reduces Aβo-induced ROS production and mitochondrial dysfunction. Therefore, RB@LCP-AR was selected for subsequent animal experiments.

### 3.3 RB@LCP-AR rescues the memory loss and recognition impairment of AD mice

**Fig. 4:**
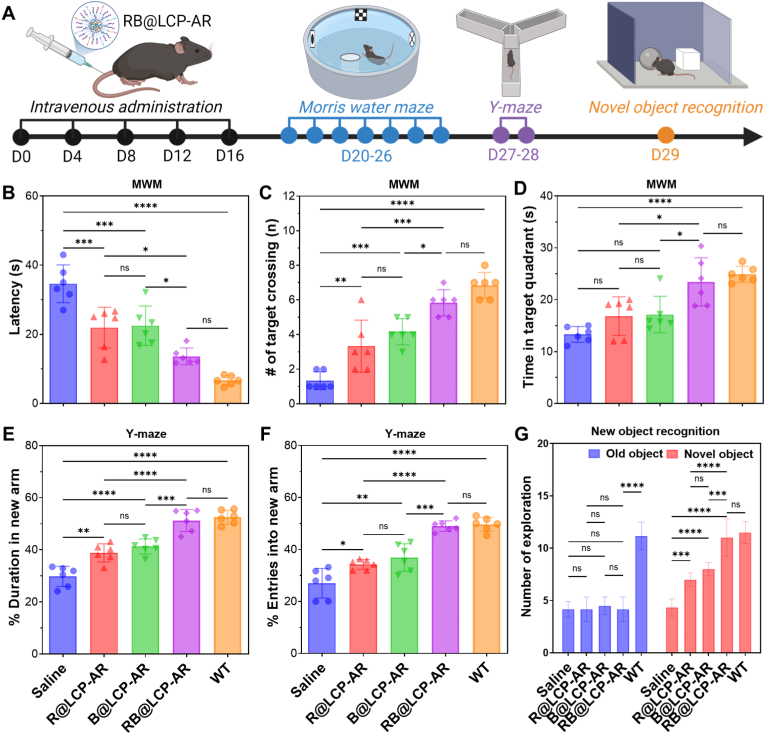
Behavior test of AD mice treated with RB@LCP-AR. (A) Timeframe of treatment and animal behavior tests. (B–D) MWM test for the latency of AD mice to the removed platform area (B), the number of platform area crossings (C), and the time spent in the target quadrant (D). (E–F) Performance in the Y-maze test was quantified as the duration spent (E) and entries into novel arm (F). (G) The recognitive preference of old and novel objects of AD mice treated with RB@LCP-AR. Data are presented as mean ± SEM (n = 6). ∗*p* < 0.05, ∗∗*p* < 0.01, ∗∗∗*p* < 0.001, ∗∗∗∗*p* < 0.0001.

The BBB penetration capacity of RB@LCP-AR was first evaluated in an *in vitro* trans-well model, which shows that both Ang and RVG modified nanoparticles effectively crossed the b.End3 cell layer located in the above chamber and reached the N2a cells located in the well (Fig. S3A). Notably, nanoparticles simultaneously modified with Ang and RVG more effectively crossed the b.End3 cell layer and finally reached to N2a cells (Fig. S3A). This is largely attributed to the expression of nAChR receptor in both b.End3 and N2a cells (Fig. S3B), which enables RVG29-modified nanoparticles cross the endothelial layer while targeting N2a cells like our previous report [40]. On this basis, the biodistribution and brain-targeting efficiency of RB@LCP-AR after intravenous injection were further evaluated. As many nanomedicines, RB@LCP-AR mainly accumulated in the liver and lung (Fig. S3C). However, surface modification with Ang significantly enhanced BBB penetration, leading to 1.9-fold higher brain MFI in the RB@LCP-AR group compared with RB@LCP (Fig. S3D) [43]. By analyzing the change of Ca^2+^ in the brain, the brain accumulation of RB@LCP-AR was estimated to be around 2.2% (Fig. S3E). Based on this enhancement, APP/PS1 AD mice were intravenously administered RB@LCP-AR (50 mg kg^−1^ per dose) 5 times at four-day intervals, followed by behavioral assessment using the MWM, Y-maze, and novel object recognition tests (Fig. 4A). In the MWM, AD mice were trained to locate a hidden platform for 6 days, after which the platform was removed on day 7. The MWM trajectories (Fig. S4) directly evidence the therapeutic efficacy of RB@LCP-AR, which restored targeted spatial navigation in AD mice, comparable to wild-type (WT) performance. RB@LCP-AR–treated mice located the former platform area within 13.6 s, which was close to that of WT mice (6.6 s), but significantly better than AD mice treated with R@LCP-AR (21.9 s) or B@LCP-AR (22.5 s) (Fig. 4B). Furthermore, RB@LCP-AR-treated AD mice showed more frequent crossings and longer time spent in the target area, similar to WT mice and markedly higher than saline-, R@LCP-AR-, or B@LCP-AR-treated AD mice (Fig. 4C and D), indicating recovery of spatial learning and memory. Consistently, Y-maze testing demonstrated that RB@LCP-AR-treated AD mice preferentially explored the novel arm, with more entries and longer residence times, comparable to WT mice but much better than other groups of AD mice (Fig. 4E and F). Likewise, in the novel object recognition test, RB@LCP-AR-treated mice more readily identified the novel object, further confirming improved memory and cognition (Fig. 4G).

Intravenous injection as a most widely used approach enables nanomedicine quickly accumulate to the targeted tissues, however, non-specific accumulation of nanomedicine in normal tissues is unavoidable. Fortunately, biosafety evaluation revealed no significant body weight loss in any treatment group (Fig. S5A). The level of serum alanine transaminase (ALT) and aspartate transaminase (AST) in mice treated with R@LCP-AR, B@LCP-AR, or RB@LCP-AR was all comparable to the saline group, indicating no apparent liver injury despite liver accumulation (Fig. S5B–C). Furthermore, analysis of inflammatory cytokines, including IL-6 and TNF-α, showed that multiple intravenous injections of the indicated formulations did not increase but rather decreased systemic inflammation compared with saline-treated AD mice (Fig. S5D–E). At the end of all behavior experiments, the organs were collected for analyzing the residual of RB@LCP-AR by measuring the content of Ca^2+^. Our results indicate that almost all RB@LCP-AR have been scavenged from the body as the level of Ca^2+^ in major organs was similar to that in saline-treated AD mice (Fig. S5F). In addition, hematoxylin and eosin (H&E) staining revealed no obvious pathological injuries in major peripheral organs, particularly the liver, lung, and kidney (Fig. S5G). Taken together, the inherent biocompatibility and biodegradability of the LCP and drug safety ensure RB@LCP-AR is a safe and effective neuroenhancer for rescuing memory loss and cognitive impairment in AD mice under multiple intravenous injection.

### 3.4 RB@LCP-AR decreases Aβ burden in AD mice

**Fig. 5:**
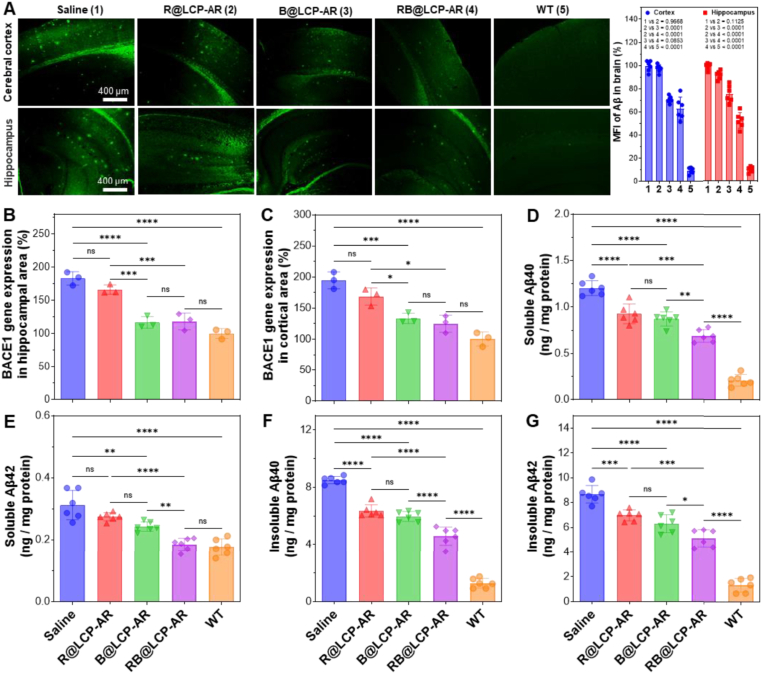
RB@LCP-AR reduces Aβ burden in brains of AD mice. (A) Distribution of Aβ plaques in the cerebral cortex and hippocampus areas of brain in AD mice treated with the indicated formulations and the corresponding MFI analysis of Aβ fluorescence. Scale bar = 400 μm. (B–C) Analysis of the BACE1 mRNA expression in the hippocampus (B) and cerebralcotrtex (C) of AD mice (n = 3). (D–G) The levels of Aβ40 and Aβ42 in soluble (D–E) and insoluble (F–G) forms in AD mouse brain (n = 6). Data are presented as mean ± SEM. ∗*p* < 0.05, ∗∗*p* < 0.01, ∗∗∗*p* < 0.001, ∗∗∗∗*p* < 0.0001.

The improvement of memory and recognition in AD mice is largely attributed to the clearance of abnormal Aβ plaques from the brain, a strategy already validated in clinical settings [44]. As shown in Fig. 5A, RB@LCP-AR treatment markedly reduced both the number of Aβ plaques and the MFI of Aβ fluorescence in the cerebral cortex and hippocampus. The synergy index confirms a synergistic effect of the RB@LCP-AR combination in reducing Aβ burden (Table S2). In comparison, R@LCP-AR did not significantly reduce Aβ burden in APP/PS1 AD mice, whereas B@LCP-AR achieved a reduction similar to RB@LCP-AR. This indicates that siBACE1, rather than Rutin, is primarily responsible for decreasing Aβ production by inhibiting BACE1-mediated APP cleavage. Consistently, BACE1 mRNA levels in the cortex and hippocampus were significantly reduced by B@LCP-AR and RB@LCP-AR, approaching that of WT mice (Fig. 5B and C). As a result, both Aβ40 and Aβ42 levels in soluble and insoluble forms were significantly decreased in the brain of treated AD mice, reflecting reduced Aβ production (Fig. 5D–G) [45]. Although R@LCP-AR did not affect BACE1 expression, it substantially lowered soluble Aβ40 and insoluble Aβ40/42 levels by binding to aggregates and promoting their clearance (Fig. 5D–G). In sharp contrast, RB@LCP-AR combined the functions of siBACE1 (Aβ production inhibition) and Rutin (Aβ clearance), and most pronouncedly reduced Aβ40 and Aβ42 levels in both soluble and insoluble forms (Fig. 5D–G). These results suggest that the synergistic action of siBACE1 and Rutin accelerates Aβ aggregate clearance from the AD brain, thereby providing the mechanistic basis for memory and cognitive recovery.

### 3.5 RB@LCP-AR reduces neuroinflammation and rescues neuronal synapse loss

**Fig. 6:**
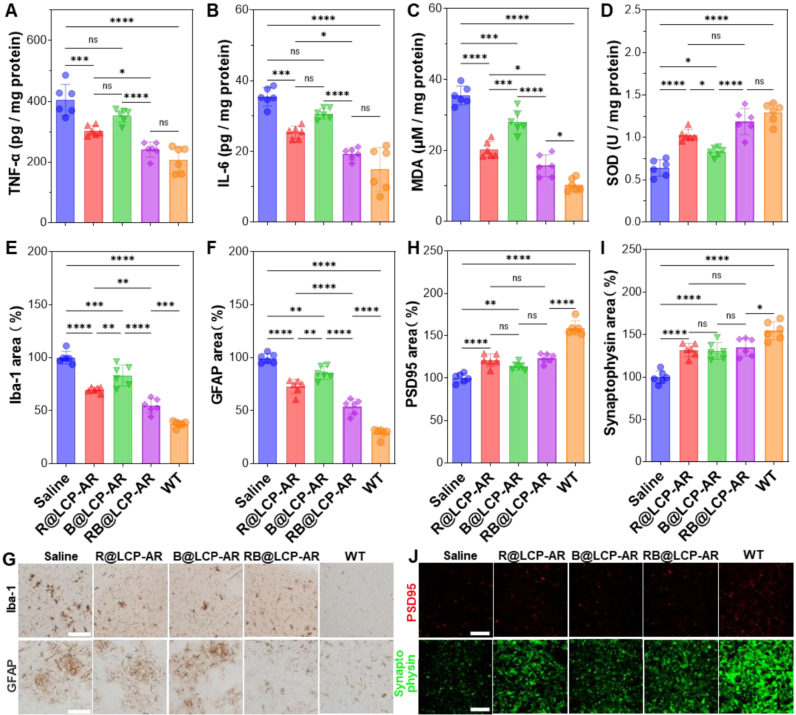
RB@LCP-AR diminishes neuroinflammation and restores neuronal synapses in AD mice. AD mice were intravenously injected with the indicated formulations 5 times, then the brain tissues were collected for further analysis. (A–B) The levels of inflammatory TNF-α (A) and IL-6 (B). (C–D) The levels of MDA (C) and SOD (D). (E–G) The distribution and quantitative analysis of Iba-1 (astrocytes) and GFAP (microglia) in the brain tissues. Scale bar = 400 μm. (H–J) The distribution and quantitative analysis of PSD95 (postsynaptic) and synaptophysin (presynaptic) in the brain tissues. Scale bar = 400 μm. Data are presented by mean ± SEM (n = 6). ∗*p* < 0.05, ∗∗*p* < 0.01, ∗∗∗*p* < 0.001, ∗∗∗∗*p* < 0.0001.

Although B@LCP-AR effectively downregulated BACE1 expression and reduced Aβ burden in the brain of AD mice, it showed very limited efficacy in alleviating neuroinflammation. Levels of proinflammatory TNF-α and IL-6 in B@LCP-AR-treated mice were comparable to that in the saline group (Fig. 6A and B). Similarly, the oxidative damage marker MDA decreased only slightly (from 35.5 μM mg^−1^ protein in saline-treated mice to 28.1 μM mg^−1^ protein in the B@LCP-AR group), while SOD activity increased only by 1.3-fold (Fig. 6C and D). These findings suggest that reducing Aβ burden *via* siBACE1 did not mitigate the established neuroinflammation induced by Aβ aggregates, a phenomenon also reported clinically [9]. In sharp contrast, R@LCP-AR significantly lowered the level of TNF-α, IL-6, and MDA but enhanced SOD activity (Fig. 6A–D). This effect was further amplified in RB@LCP-AR–treated mice, where TNF-α, IL-6, and MDA were reduced by ∼50–65% compared with saline, and SOD activity increased by 1.8-fold (Fig. 6A–D). These results highlight that the combination of siBACE1-mediated Aβ reduction and Rutin-mediated anti-inflammation effectively alleviates the neuroinflammation caused by Aβ burden and nanoparticle clearance in the AD brain.

The reduction of Aβ burden also contributed to decreased astrogliosis and microgliosis, the key drivers of neuroinflammation in AD [46]. Clearly, RB@LCP-AR treatment markedly decreased Iba-1 (microglial marker) and GFAP (astrocytic marker) levels (by 45% and 52%) compared with the saline group (Fig. 6E–G), attributable to Rutin's anti-inflammatory activity, which mitigated glial overactivation during nanoparticle clearance. Consequently, RB@LCP-AR treatment induced the highest expression of PSD95, a major synaptic scaffolding protein essential for synaptic plasticity, and synaptophysin, a key vesicle membrane protein involved in synaptic recycling and exocytosis, in the brain of AD mice (Fig. 6H–J), demonstrating that the normalization of astrocytes and microglia alleviated aberrant glial repair of neuronal protrusions [47]. Quantitative analysis revealed that the combination of R@LCP-AR and B@LCP-AR in RB@LCP-AR exhibited a synergistic effect (synergy index >1) on reducing Iba-1 and GFAP levels as well as on increasing PSD95 expression. Notably, a stronger synergy (synergy index >1.2) was observed specifically in enhancing the synaptophysin area (Table S2). Mechanistically, Rutin suppresses neuroinflammation by promoting a metabolic shift in microglia toward oxidative phosphorylation, thereby enhancing Aβ phagocytosis while limiting proinflammatory activation [48]. Consistent with this mechanism, RB@LCP-AR treatment markedly reduced proinflammatory cytokines, oxidative stress, astrogliosis, and microgliosis, while restoring synaptic markers and cognitive function.

In this research, we designed the dual-functional neuroenhancer RB@LCP-AR, co-loaded with siBACE1 and Rutin to simultaneously target Aβ production and neuroinflammation. Within neurons, siBACE1 silences BACE1 expression, reducing Aβ production by inhibiting BACE1-mediated APP cleavage. Concurrently, Rutin alleviates Aβ oligomer-induced mitochondrial dysfunction in neurons (Fig. 3). During clearance of RB@LCP-AR by microglia and astrocytes [49], Rutin further mitigates astrogliosis and microgliosis, reducing the notorious neuroinflammation. This dual action has effectively alleviated both Aβ burden and neuroinflammation in the AD mouse brain (Fig. 5, Fig. 6), restoring neuronal function and improving memory and cognition (Fig. 4).

The clinical success of FDA-approved monoclonal antibodies targeting Aβ clearance as an effective AD strategy [7,8]. However, neuroinflammation, initially induced by Aβ aggregates, can persist and progress independently during the middle and late stages of AD. RB@LCP-AR offers several advantages over conventional antibodies. First, it enhances the bioavailability of RNA and poorly soluble anti-inflammatory compounds that would otherwise be rapidly cleared. Secondly, its small particle size, together with surface-anchored Ang and RVG peptides, enables efficient BBB penetration and neuronal targeting. In the AD mouse brain, RB@LCP-AR is predominantly internalized by neurons with minimal uptake by microglia, where it rapidly escapes endosomes and releases both siBACE1 and Rutin into the cytosol [50]. Unlike antibodies that only eliminate extracellular Aβ aggregates, siBACE1 released from RB@LCP-AR effectively suppresses Aβ production by silencing BACE1, thereby reducing Aβ accumulation in both intracellular and extracellular compartments and ultimately decreasing overall plaque burden. Concurrently, Rutin mitigates mitochondrial dysfunction by scavenging ROS and inhibiting intracellular Aβ aggregation and its associated cytotoxicity [36]. Notably, excessive Aβ binding to complement component C1q triggers microglial overactivation and synaptic phagocytosis, accelerating AD progression [51]. Rutin released from RB@LCP-AR internalized by microglia alleviates this overactivation, thereby promoting neuronal restoration and improving memory and cognitive function in AD mice. Collectively, these findings emphasize that targeting neuroinflammation is essential alongside Aβ clearance, providing a strong rationale for developing next-generation AD therapeutics with synergistic multi-target capabilities.

## 4 Conclusion

The tailored RB@LCP-AR neuroenhancer simultaneously modulates Aβ generation and clearance through the co-delivery of siBACE1 and the natural compound Rutin. siBACE1 efficiently silences BACE1 expression to inhibit Aβ production, while Rutin alleviates neuronal mitochondrial dysfunction and prevents immune overactivation induced by Aβ clearance, thereby mitigating neuroinflammation. Consequently, RB@LCP-AR treatment promoted neuronal synapse regeneration and restored memory and cognitive function in AD mice. This study introduces a new paradigm for addressing persistent neuroinflammation beyond Aβ-targeted therapies, offering a promising strategy for comprehensive Alzheimer's disease treatment. The clinical translation of RB@LCP-AR is currently limited by its invasive administration route and unverified long-term systemic safety. Future studies could further validate the versatility of this LCP platform by applying it to additional therapeutic targets.

## Ethics approval and consent to participate

All animal studies were approved by the Institutional Animal Care and Use Committee of Zhejiang University (AP CODE: ZJU20220226).

## Consent for publication

All authors of this study agreed to publish.

## Funding

This work was financially supported by the 10.13039/501100000780European Union’s Research and Innovation Program under the 10.13039/100010665Marie Skłodowska-Curie grant agreement (No. 101064861), the 10.13039/501100001809National Natural Science Foundation of China (No. 82101591), the 10.13039/501100004731Natural Science Foundation of Zhejiang Province (No. LQ20H090014), the Natural Science Foundation of Hangzhou Municipality (2025SZRJJ1086), the Queensland-10.13039/501100002367Chinese Academy of Sciences Collaborative Science Fund (Grants 122111KYSB20180005 and QCAS02017-18RD7), and Hangzhou City University Strating Grant (to L.Z.).

## CRediT authorship contribution statement

**Weiqing Fang:** Formal analysis, Funding acquisition, Investigation, Methodology, Writing – original draft. **Jing Zhao:** Formal analysis, Investigation, Methodology, Writing – original draft. **Li Li:** Formal analysis, Investigation, Methodology, Writing – original draft. **Yu Wang:** Investigation, Methodology. **Zhi Ping Xu:** Conceptualization, Funding acquisition, Supervision, Writing – review & editing. **Lingxiao Zhang:** Conceptualization, Funding acquisition, Investigation, Project administration, Supervision, Writing – review & editing.

## Declaration of competing interest

The authors declare that they have no known competing financial interests or personal relationships that could have appeared to influence the work reported in this paper.

## Data Availability

Data will be made available on request.
