# Supplementary material for: An anti-inflammatory neuroenhancer mitigates amyloid-β pathology to improve Alzheimer's disease therapy
Source: Mater Today Bio. 2026 Jan 31;37:102874. doi: 10.1016/j.mtbio.2026.102874 (PMC12906037; doi:10.1016/j.mtbio.2026.102874)
Supplement: Multimedia component 1 [file mmc1.docx]

**Supplementary information**

**An anti-inflammatory neuroenhancer mitigates amyloid-β pathology to improve Alzheimer’s disease therapy**

*Weiqing Fang^1,a^, Jing Zhao^2,3,a^, Li Li^4,a^, Yu Wang^2^, Zhi Ping Xu^2,3^, Lingxiao Zhang^2,3,5,*^*

^1^ Department of Pharmacy, School of Medicine, Women’s Hospital, Zhejiang University, Hangzhou 310023, China.

^2^ School of Medicine, Hangzhou City University, Hangzhou 310015, China.

^3^ Institute of Chemical Biology, Shenzhen Bay Laboratory, Shenzhen 518107, China.

^4^ Gulbali Research Institute, Charles Sturt University, Wagga NSW 2678, Australia.

^5^ Interdisciplinary Nanoscience Center, Aarhus University, Aarhus C DK-8000, Denmark.

^a^ These authors contribute equally.

***Corresponding author.**

A/Prof. Lingxiao Zhang, *E*-mail: [zhanglx@inano.au.dk](mailto:zhanglx@inano.au.dk)

**Table S1. Composition and component amount of RB@LCP-AR**

| **Sample** | **Abbreviation** | **LCP** | **Rutin** | **siBACE1** | **Ang** | **RVG** |
| --- | --- | --- | --- | --- | --- | --- |
| Rutin@LCP | R@LCP | 1 mg | 118 μg | / | / | / |
| siBACE1@LCP | B@LCP | 1 mg | / | 24 μg | / | / |
| Rutin/siBACE1@LCP | RB@LCP | 1 mg | 118 μg | 24 μg | / | / |
| Rutin/siBACE1@LCP-Ang/RVG | RB@LCP-AR | 1 mg | 118 μg | 1. μg | 24 μg | 34 μg |

**Table S2. Combined effect of R@LCP-AR and B@LCP-AR**

| **Target**  **marker** | **R@LCP-AR (A)**  **%** | **B@LCP-AR (B)**  **%** | **RB@LCP-AR (C)**  **%** | **Synergy index [A]×[B]/[C]** |
| --- | --- | --- | --- | --- |
| Aβ plaques in cerebral cortex | 98.0 ± 3.3 | 71.2 ± 3.0 | 63.3 ± 9.8 | 1.12 ± 0.17 |
| Aβ plaques in hippocampus | 92.5 ± 3.7 | 76.1 ± 7.5 | 52.8 ± 6.8 | 1.35 ± 0.18 |
| Iba-1 | 69.8 ± 2.6 | 83.2± 10.0 | 54.6 ± 6.8 | 1.08 ± 0.14 |
| GFAP | 73.3 ± 6.7 | 86.0 ± 6.2 | 54.3 ± 7.2 | 1.18 ± 0.17 |
| PSD95 | 120.7 ± 7.4 | 113.7 ± 4.3 | 123.3 ± 5.4 | 1.11 ± 0.05 |
| Synaptophysin area | 131.9 ± 7.6 | 130.9 ± 9.9 | 135.4 ± 10.7 | 1.28 ± 0.1 |

[A] = Marker expression (R@LCP-AR) / Marker expression (Saline)×100%;

[B] = Marker expression (B@LCP-AR) / Marker expression (Saline)×100%;

[C] = [A+B] = Marker expression (RB@LCP-AR) / Marker expression (Saline)×100%;

The synergy index was calculated as [A]×[B]/[C], and the value > 1.2 was thought to be significant.


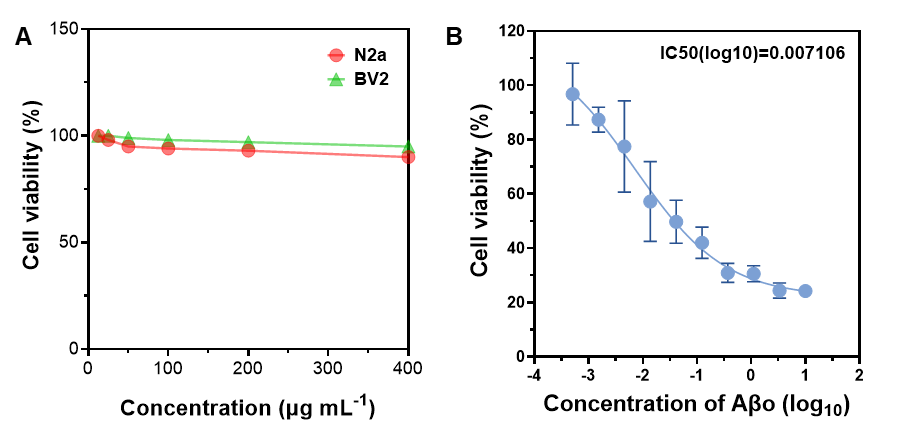


**Fig. S1. Cell viability.** (A) Both N2a and BV2 cells were treated with the RB@LCP-AR at a concentration range from 12.5 to 400 μg mL^-1^ for 24 h, then the cell viability was determined by MTT assay. (B) N2a cells treated with different concentrations of Aβo (0.0001-10 μM). Data are presented as mean ± SEM (n = 6).


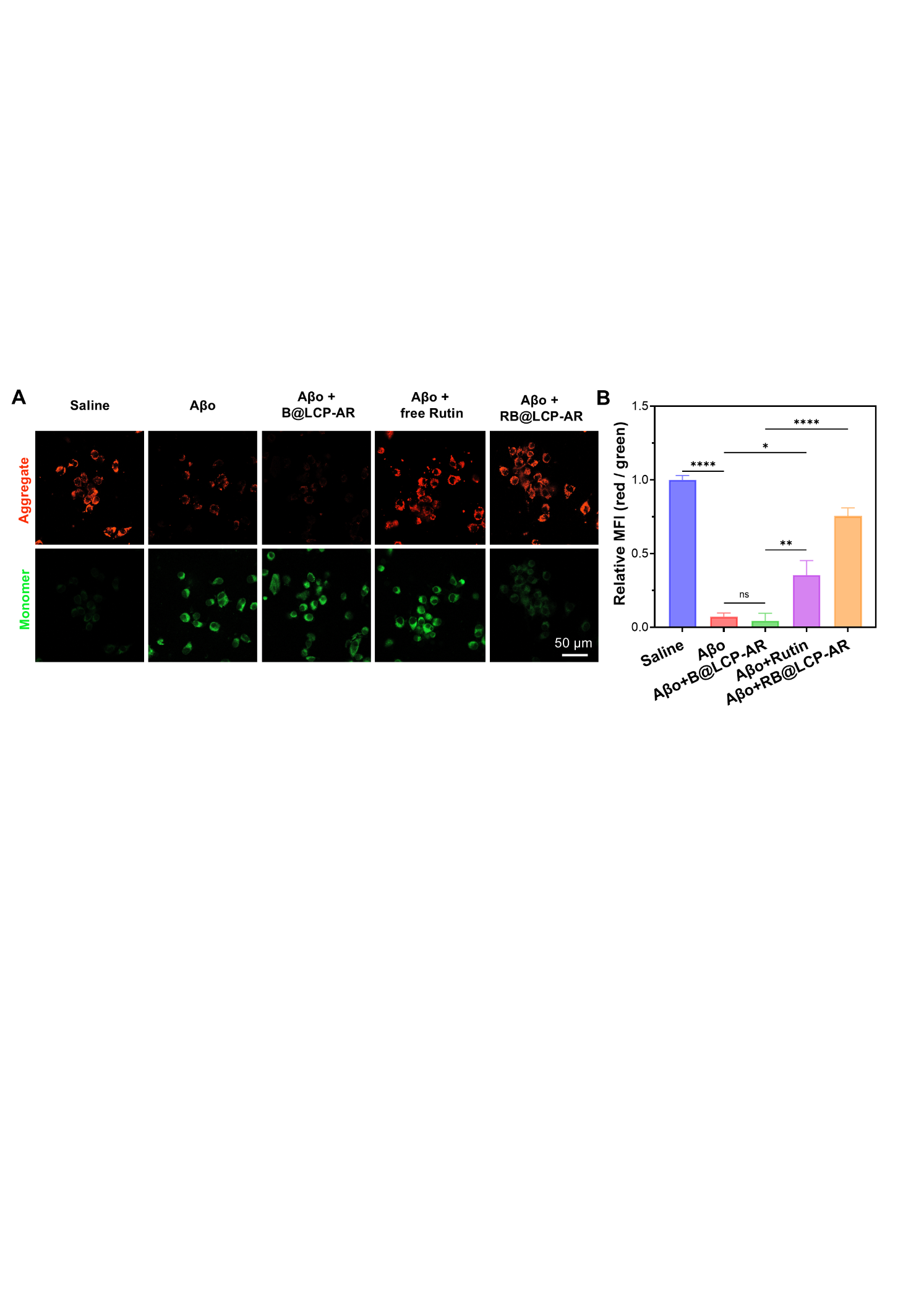


**Fig. S2 Reduction of Aβo-induced ROS and mitochondrial dysfunction by RB@LCP-AR.** N2a cells treated with Aβo (1 μM) and the indicated formulations (Rutin 11.8 μg mL^-1^, B@LCP-AR or RB@LCP-AR: 100 μg mL^-1^) were stained with JC-1, then distribution of JC-1 aggregate and monomer was imaged by confocal laser scattering microscopy (A), and the ratio of red to green fluorescence intensity was calculated (B). Data are presented as mean ± SEM. **p* < 0.05, ***p* < 0.01, ****p* < 0.001, *****p* < 0.0001.


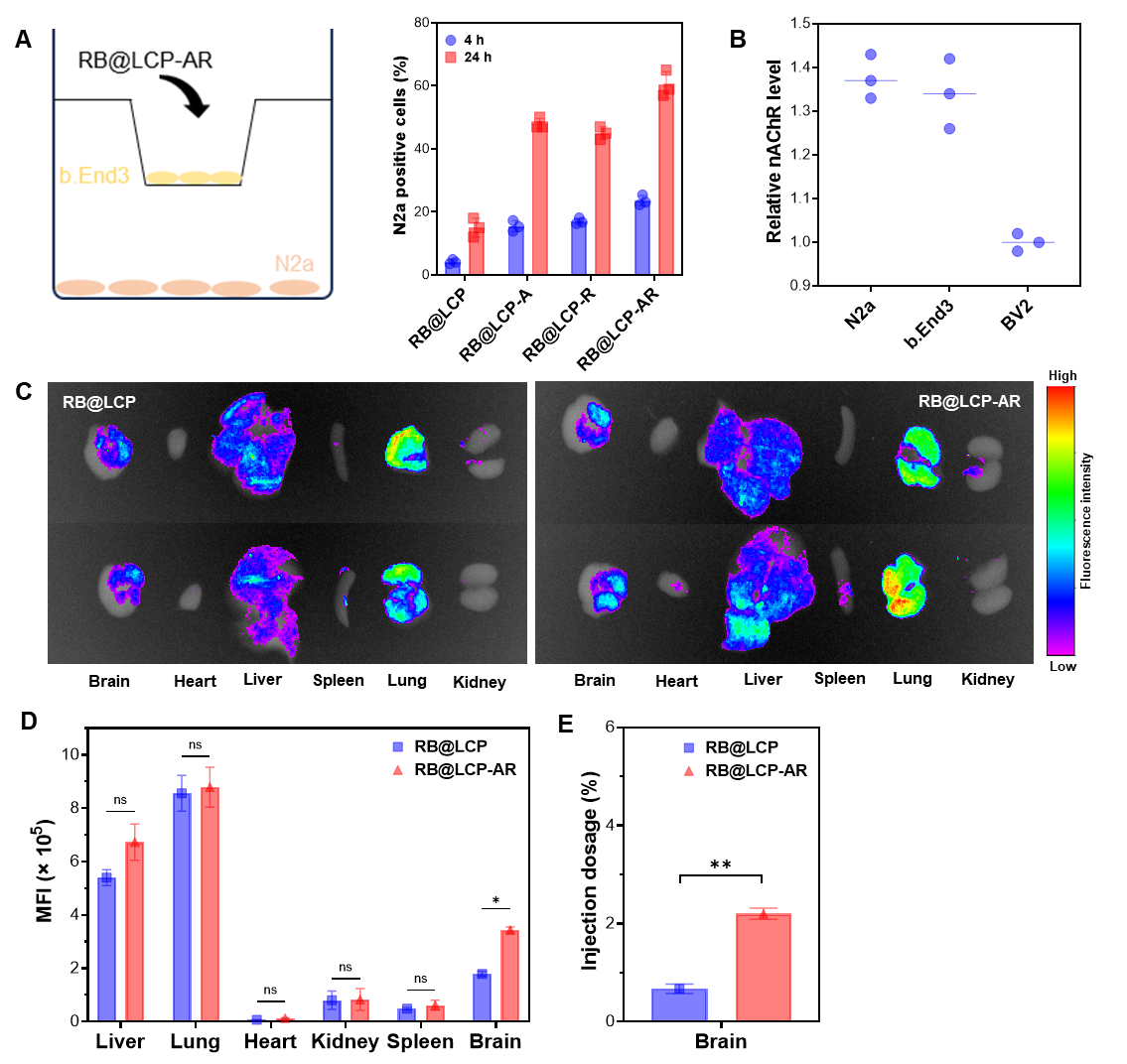


**Fig. S3 Biodistribution of RB@LCP-AR.** (A) Schematic illustration of the BBB-mimic penetration trans-well experiment. The indicated Cy5-labeled nanoparticles were then added into the above chamber, and the proportion of positive N2a cells in the well was analyzed by flow cytometry at 4 and 24 h. (B) Expression of nAChR gene in the indicated cells were analyzed by q-PCR (n=3). (C) Biodistribution of RB@LCP and RB@LCP-AR (Cy5 was loaded in the core of LCP) in major organs of AD mice at 24 h after intravenous injection (n = 3). (D) Quantification of the MFI of LCP in the major organs of (A). (E) Accumulation of the indicated nanoparticles in the brain of AD mice were estimated by comparing the total Ca^2+^ amount in the brain tissues to the injected dosage through inductively coupled plasma mass spectrometry. AD mice without treatment was used a control to remove the basic Ca^2+^ content in the brain. Data are presented as mean ± SEM. **p* < 0.05, ***p* < 0.01, ****p* < 0.001, *****p* < 0.0001.

**
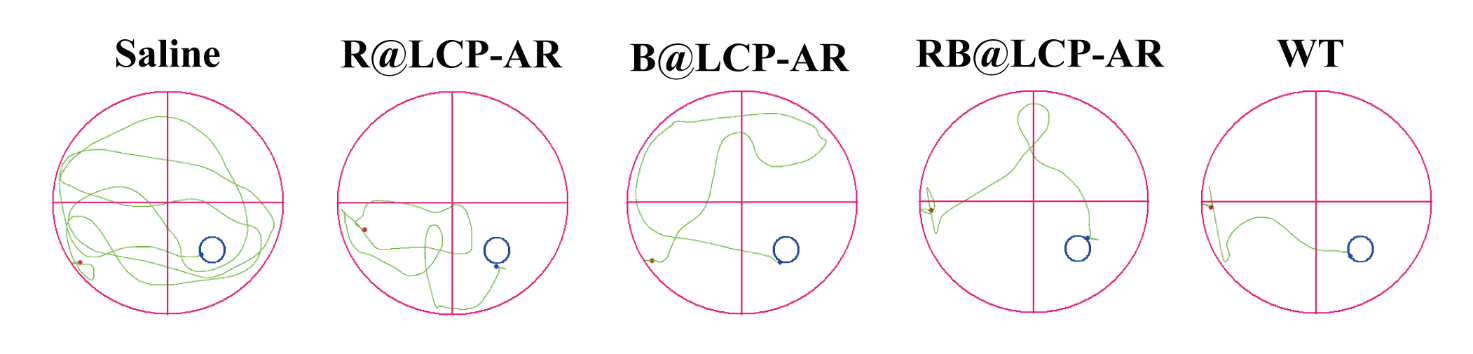
**

**Fig. S4 Representative Morris water maze swimming trajectories following the indicated treatment.**


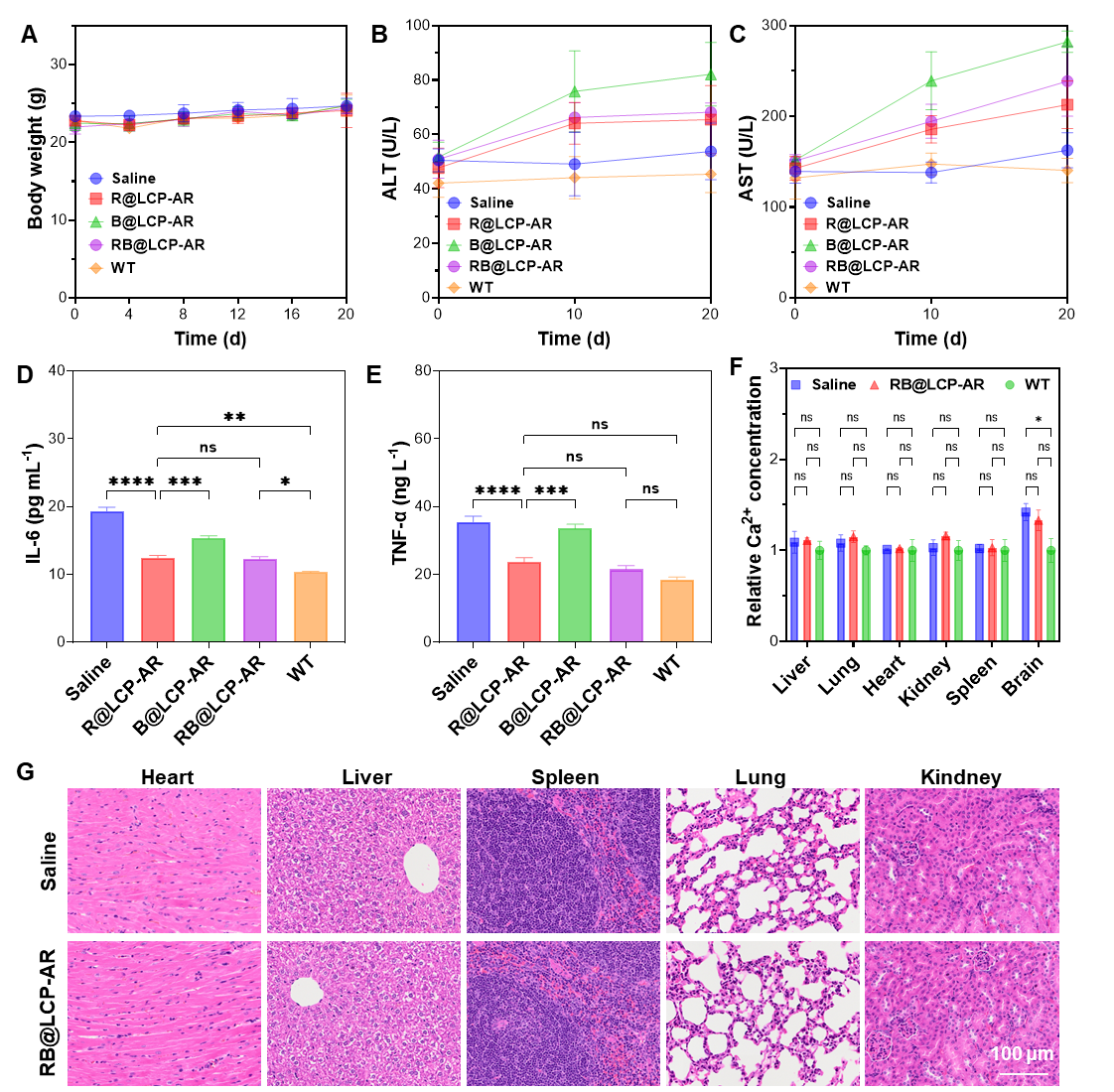


**Fig. S5 Biosafety of the formulations.** AD and WT mice were intravenously injected with the indicated formulations at an interval of 4 days during the 20-day treatment period. (A) The body weight of mice was recorded before each injection. (B-C) The levels of alanine aminotransferase (ALT) and aspartate aminotransferase (AST) in serum of mice were detected at day 0, 10 and 20. (D-E) Levels of IL-6 (D) and TNF-α (E) in serum of AD and WT mice at day 20. (F) Relative concentration of Ca^2+^ in major organs of AD mice at 30 days after treatment (denoted in Fig. 4A) was detected by inductively coupled plasma mass spectrometry. (G) Representative H&E images of major organs at day 30. Data are presented as mean ± SEM (n = 6).
